# Pediatric head injury guideline use in Sweden: a cross-sectional survey on determinants for successful implementation of a clinical practice guideline

**DOI:** 10.1186/s12913-024-11423-z

**Published:** 2024-08-21

**Authors:** Fredrik Wickbom, William Berghog, Susanne Bernhardsson, Linda Persson, Stefan Kunkel, Johan Undén

**Affiliations:** 1 Department of Operation and Intensive Care, Halland Hospital, Halmstad, Sweden; 2 Lund University, Lund, Sweden; 3 Region Västra Götaland, Research, Education, Development, and Innovation Primary Health Care, Gothenburg, Sweden; 4 School of Public Health and Community Medicine, University of Gothenburg, Sahlgrenska Academy, Gothenburg, Sweden; 5 Institute of Neuroscience and Physiology, Department of Health and Rehabilitation, Unit of Physiotherapy, University of Gothenburg, Sahlgrenska Academy, Gothenburg, Sweden; 6 Department of Orthopedics, Halland Hospital, Halmstad, Sweden; 7 Department of Medicine, Växjö Hospital, Växjö, Sweden

**Keywords:** mTBI, Guidelines, Children, Sweden, Implementation

## Abstract

**Background:**

The Scandinavian Neurotrauma Committee guideline (SNC-16) was developed and published in 2016, to aid clinicians in management of pediatric head injuries in Scandinavian emergency departments (ED). The objective of this study was to explore determinants for use of the SNC-16 guideline by Swedish ED physicians.

**Methods:**

This is a nationwide, cross-sectional, web-based survey in Sweden. Using modified snowball sampling, physicians managing children in the ED were invited via e-mail to complete the validated Clinician Guideline Determinants Questionnaire between February and May, 2023. Baseline data, data on enablers and barriers for use of the SNC-16 guideline, and preferred routes for implementation and access of guidelines in general were collected and analyzed descriptively and exploratory with Chi-square and Fisher's tests.

**Results:**

Of 595 invitations, 198 emergency physicians completed the survey (effective response rate 33.3%). There was a high reported use of the SNC-16 guideline (149/195; 76.4%) and a strong belief in its benefits for the patients (188/197; 95.4% agreement). Respondents generally agreed with the guideline's content (187/197; 94.9%) and found it easy to use and navigate (188/197; 95.4%). Some respondents (53/197; 26.9%) perceived a lack of organizational support needed to use the guideline. Implementation tools may be improved as only 58.9% (116/197) agreed that the guideline includes such. Only 37.6% (74/197) of the respondents agreed that the guideline clearly describes the underlying evidence supporting the recommendation. Most respondents prefer to consult colleagues (178/198; 89.9%) and guidelines (149/198; 75.3%) to gain knowledge to guide clinical decision making. Four types of enablers for guideline use emerged from free-text answers: *ease of use and implementation, alignment with local guidelines and practice, advantages for stakeholders,* and *practicality and accessibility.* Barriers for guideline use were manifested as: *organizational challenges, medical concerns*, and *practical concerns.*

**Conclusions:**

The findings suggest high self-reported use of the SNC-16 guideline among Swedish ED physicians. In updated versions of the guideline, focus on improving implementation tools and descriptions of the underlying evidence may further facilitate adoption and adherence. Measures to improve organizational support for guideline use and involvement of patient representatives should also be considered.

**Supplementary Information:**

The online version contains supplementary material available at 10.1186/s12913-024-11423-z.

## Contributions to the literature

- The pediatric Scandinavian Neurotrauma Committee head injury guideline from 2016 seems well known and well used by Swedish emergency department physicians, despite lack of formal implementation.
- The study identified guideline implementation determinants that need to be addressed in both future guideline versions and in implementation strategies.
- This study contributes reference data for the Clinician Guideline Determinants Questionnaire; a novel, validated tool for assessment of determinants for guideline use, with different results compared to previous reports utilizing the questionnaire.

## Background

Head trauma is a common cause to seek emergency department (ED) care among children in Sweden. In 2022, over 33,000 cases of head injury were registered in Sweden in children 0–17 years of age, according to the Swedish National Board of Health and Welfare [1]. Of these, 22.3% were diagnosed with an intracranial injury of varying severity (including concussion), yielding an overall incidence of 1521/100 000 patients with head injuries and an incidence of 340/100 000 patients with intracranial injury. Mild traumatic brain injury (mTBI) constitutes more than 80% of pediatric TBI cases globally [2]. Most of these injured children will recover without the need for acute intervention, e.g., neurosurgery or intensive care admission [2–5].

Cranial computed tomography (CT) utilizes ionizing radiation for imaging of the brain and is a valuable tool for excluding significant intracranial injuries, ordered in 4% of children with isolated head trauma in southern Sweden [6]. Radiation exposure in early life entails a risk of malignancy development later in life, and the selection of patients with mTBI for neuroimaging poses a clinical challenge [5, 7–9]. Structured in-hospital observation is considered equally effective, although this is associated with higher resource use [10, 11]. In Sweden (and similar to other countries), it is often junior physicians who initially manage these children, following a diverse range of local guidelines (or no guideline), resulting in an unstandardized approach to pediatric TBI on a national level [12, 13].

The Scandinavian Neurotrauma Committee has recently developed a clinical practice guideline addressing the initial management of mTBI in children (SNC-16 guideline) in Scandinavia [14]. It was published in 2016 and has since then been passively disseminated into more than 50% of the Swedish emergency hospitals’ management routines [13]. Although validated in other settings, the SNC-16 guideline has not been validated in the Scandinavian population [15, 16]. The SNC-16 guideline for managing patients with mTBI has been developed to help healthcare providers make informed management decisions. To assess the risk of intracranial injury, various factors such as clinical signs and symptoms (e.g., loss of consciousness, amnesia, neurological deficits) and current state of consciousness are considered in the guideline. If a patient's clinical status falls within the low-risk criteria, a CT scan or prolonged structured observation may be deemed unnecessary [14].

The process of clinically adapting research-based knowledge is widely acknowledged as intricate and non-self-regulating [17–19]. Clinical practice guidelines are considered valuable tools for integrating the latest medical evidence into clinical practice [20, 21]. By identifying existing barriers and facilitators that influence the use of specific guidelines, it may be possible to tailor an implementation process and facilitate the uptake of a guideline into clinical settings and ensure adequate compliance [19, 22, 23].

In 2019, the Clinician Guideline Determinants Questionnaire (CGDQ) was developed and published by Gagliardi et al. [24]. This tool serves the purpose of providing a comprehensive and validated instrument for addressing factors relevant for the use or non-use of a specific guideline from a clinician's perspective. Knowledge about determinants for use and non-use specific for the SNC-16 guideline may support an implementation process and increase adherence to evidence-based practices in managing pediatric head trauma in Sweden. It may also give important information in future updates of the guideline.

The primary objective of this study was to identify barriers and enablers affecting use of the SNC-16 guideline by physicians in Sweden. Knowledge about these determinants is important as it allows development of tailored interventions in forthcoming implementation processes with the intention to promote uptake of research findings in routine care [24]. This study is part of a series of studies which embraces validation, development, and implementation of the SNC-16 guideline in Scandinavia.

## Methods

### Study design

This is a cross-sectional observational study in Sweden. Collection of data was performed using a validated questionnaire for implementation research [24]. Respondents were asked to assess the SNC-16 guideline based on the structured questions in the questionnaire. Reporting follows STROBE guidelines for cross-sectional studies (Additional file 1) [25]. An ethical advisory opinion was granted by the Swedish Ethical Review Authority (Dnr 2020 – 02 693).

### Setting

The survey was sent to physicians in Swedish EDs of varying sizes nationwide, in which head trauma in pediatric patients is managed. Data were collected during February 23 to May 8, 2023.

### Participants

Physicians from various medical specialties who regularly, at their own discretion, work in the ED of a Swedish hospital and assess pediatric acute head trauma, were included. Respondents not fulfilling the above criteria were excluded.

Potential participants were invited by an e-mail containing an information text and a link to the questionnaire. The initial e-mail recipient list of potential respondents was based on three different e-mail collection strategies: 1) a list of suggested respondents from a previous study, investigating management of pediatric TBI in Sweden at an organizational level [13]; 2) new e-mails to ED managers with a request to send us e-mail addresses to ED physicians working with pediatric mTBI in their ED (as the list from 2022 may contain irrelevant recipients or old e-mail addresses); and 3) screening of e-mail recipient lists accessible for our research team (identifying physicians in the department of general surgery in the Region of Halland, physicians in the department of emergency medicine in the Region of Halland and interns employed in the Region of Halland, Sweden). Only potential e-mail recipients suggested from a hospital that managed children with pediatric head trauma were included when extracting the e-mail list, drawn from the 66 hospitals included in the 2022 paper (370 e-mail addresses).

**Fig. 1:**
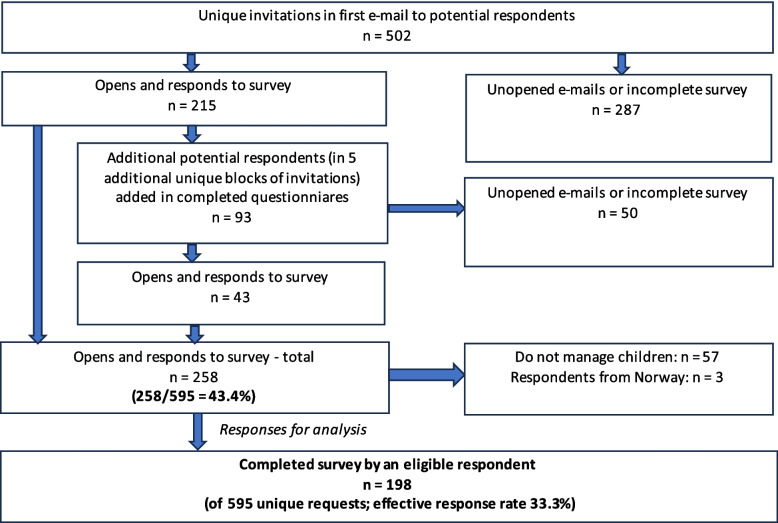
Flowchart describing structure for collection of the final data set

In summary, the final e-mail recipient list in the first block contained 502 unique e-mail addresses to potential respondents (Fig. 1). Non-responders were sent a total of five reminders during the time for data collection.

Before completing the survey, participants were asked to contribute with e-mail addresses to additional colleagues in their hospital or neighboring hospitals who they believed fulfilled the above inclusion criteria. Respondents not fulfilling the inclusion criteria were given the option to decline participation but still contribute with e-mail addresses to suitable colleagues. New e-mail addresses were added in blocks and generated in total five consecutive groups with new e-mail addresses to whom the survey was distributed. With this modified snowball sampling method, it was possible to control response rates. The study size was reached when no more new e-mail addresses were added by respondents with the snowball method, and no more non-respondents answered the survey despite multiple reminders. Respondents were pseudonymized at analysis and no patient data was recorded.

Respondents are by definition fluent in both Swedish and English as this is a criterion for admission to medical training in Swedish universities and hospitals. The medical literature in Sweden is also predominately in English.

### Measures

The Clinician Guideline Determinants Questionnaire (CGDQ) was used for data collection [24]. It is a validated instrument for preparing and evaluating implementation of clinical practice guidelines. The CGDQ includes four sections exploring: 1) clinician demographic and background information; 2) attitudes to known determinants of guideline use; 3) open-ended items on additional determinants; and 4) a section examining preferred ways of distribution, access, and character of a guideline. The CGDQ was transcripted unchanged from the original version and presented in English in a digital questionnaire in the web-based survey system EsMaker (Entergate AB). As respondents have a high knowledge of the English language, we judged the risks associated with a translation of the questionnaire to Swedish greater than the risk that respondents would not understand the questions. Three questions exploring what size and type of hospital the respondent worked in, type of patients (children/adults/both) they managed, and their familiarity with assessing children with head injury were added to the background information section by the authors. The SNC-16 flow chart, a link to the original publication, and a link to an article in the Swedish medical journal Läkartidningen were presented at the beginning of the questionnaire [14, 26]. The text “SNC-16 guideline” was inserted in the questionnaire where stated, “name guideline”. Some items have been truncated to improve readability in the results section of this paper, with a reference to the full questionnaire and complete items in Additional file 2.

### Bias

To minimize the risk for introducing selection bias, purposive sampling was used to include respondents from varying parts of Sweden and from varying hospital sizes, and including both junior and senior physicians, when compiling the initial respondent mailing list.

### Data analysis

Reported data are categorical nominal/dichotomous or categorical ordinal (on a 7-step Likert scale, including response option “not sure”), or in free text. Responses to categorical nominal items are summarized and presented as frequencies and percentages. Variables that are reported on an ordinal 7-step Likert scale were dichotomized into “disagree” if Likert response 1–4 or unsure, and into “agree” if Likert response 5–7. The unmerged response distribution is shown in Additional file 3. Results are presented for the four sections in the applied implementation tool (CGDQ). Merging of categories was performed if there were few responses in a response category.

Background data on respondents are presented descriptively for a) gender, b) career stage (as found most appropriate by the respondent), c) medical specialty, d) hospital category (local hospital, regional hospital, university hospital or children’s hospital – with local and regional merged as small hospitals and university and children’s as large), e) region in Sweden, f) managing only children or both children and adults, g) familiarity with assessing children with head injury (categorized as “daily” + “several times a week” = regularly; “1–3 times/month” = seldom; “5–10 times/year” + “1–4 times/year” + “less than once a year” = rarely), h) have participated in the development of one or more guidelines, i) belief in clinical benefit of guidelines, and j) actual use of SNC-16 guideline.

Frequencies and percentages for "agree” and “disagree” for determinants in Sect. 2 of the survey were calculated. The authors decided to perform further analysis on a subset of factors from the clinician and guideline specific determinants in Sect. 2, aiming to explore possible associations between determinants and background factors. The subset comprised six variables selected by the authors after reviewing initial results and considered most salient to grasp the respondent’s thoughts on the guideline and their knowledge about the relevant clinical condition, with the most clinically relevant imprint. Authors decided to not test all items as it would entail an unjustified risk for significant results by chance. Chi-square test, or Fisher’s exact test when appropriate, was used to assess associations.

The free-text responses obtained from questions 3.1 to 3.4 (additional file 2) were independently categorized into types of barriers and enablers by two of the authors (FW, WB) and then compiled in consensus.

## Results

### Participants

The first invitation e-mail was sent on February 23, 2023. The final reminder was sent on April 20, 2023. Respondents suggested 93 additional unique potential respondents, resulting in invitations also sent to these individuals. In this group, 43 participants opened the e-mail and participated in the survey, yielding a response rate in the snowball sample group of 46.2%. The total response rate was 43.4% (258/595; opens and responds to request) with an effective response rate for analysable respondents of 33.3% (198/595) (Fig. 1).

### Background information

The 198 responding physicians from 42 unique EDs had varying clinical experience, in a span from early career interns (14.1%; 28/198), mid-career residents (48.5%; 96/198), to late career consultants (37.4%; 74/198). The most common specialties represented were general surgery (52.0%; 103/198) and emergency medicine (31.8%; 63/198). A majority (82.3%; 163/198) of the respondents worked in small (local or regional hospitals) compared to 17.7% (*n* = 35) in large (university or children’s) hospitals. There was a high degree of familiarity with the SNC-16 guideline, as 84.3% (166/197) had “read all or some of the guideline on multiple occasions” and only 8.1% (16/197) were unaware of the guideline or “aware of the guideline but have not read it”. A high proportion (76.4%; 149/195) of respondents reported regular use of the SNC-16 guideline in their respective clinical settings, and almost all (95.4%; 188/197) believed that guideline use in general optimized healthcare delivery and outcomes (Table 1).

**Table 1:** Participant characteristics

	**No**	**%**
**Gender (*n* = 196)**		
Male	99	50.5%
Female	96	49.0%
Prefer not to respond	1	0.5%
**Career stage (*n* = 198)**		
Early (Intern)	28	14.1%
Mid (Residency)	96	48.5%
Late (Consultant)	74	37.4%
**Specialty (*n *= 198)**		
Pediatric medicine	17	8.6%
General surgery	103	52.0%
Emergency medicine	63	31.8%
Other^a^	31	15.7%
**Category of hospital (*n* = 198)**		
Children’s	9	4.5%
University	26	13.1%
Local	32	16.2%
Regional	131	66.2%
* Small (local and regional)*	*163*	*82.3%*
* Large (university and children’s)*	*35*	*17.7%*
**Part of Sweden (*n* = 198)**		
Southern	145	73.2%
Central	36	18.2%
Northern	17	8.6%
**Types of patients managed in respondents ED (*n* = 198)**		
Children	24	12.1%
Children and adults	173	87.4%
Adults	1	0.5%
**Frequency of assessing children with mild head injury (*n* = 198)**		
Daily	13	6.6%
Several times per week	74	37.4%
1–3 times/month	96	48.5%
5–10 times/year	10	5.1%
1–4 times/year	5	2.5%
**I believe that guidelines (in general) optimize health care delivery and outcomes… (*n* = 197)**		
Yes	188	95.4%
No	1	0.5%
Unsure	8	4.1%
**I have participated in guideline development of one or more guidelines (*n* = 197)**		
Yes	66	33.5%
No	131	66.5%
**What is your intended or actual use of the SNC-16 guideline? (*n* = 195)**^b^		
Regularly	149	76.4%
Not regularly	46	23.6%

^a^Other specialties = pediatric surgery (*n* = 9), internal medicine (*n* = 4), orthopedics (*n* = 5) and other (*n* = 13; urology/primary care/pediatric cardiology/pediatric emergency medicine/intern/anesthesia). As this was a multiple-choice question, the sum is not *n* = 197)

^b^One item from the determinants of guideline use section was deemed of certain importance as it may influence responses in other domains and is therefore reported descriptively in Table 1. Not regularly is the merged response rate of “never used the guideline…” and “have used the guideline once” or”…a few times”. See additional file 3 for original response distribution

### Determinants of guideline use

It was common among respondents to think that colleagues (77.8%; 154/198) expected them to use the SNC-16 guideline. Fewer believed that patients (12.1%; 24/198), managers/executives in their own organization (37.9%; 75/198), a monitoring agency (Swedish National Board of Health and Welfare: 15.7%; 31/198), the government (4.0%; 8/198), and/or the professional society (23.7%; 47/198) expected them to use the guideline.

The attitude towards use of the SNC-16 guideline was generally positive as 94.9% (187/197) agreed with the content of the guideline. Approximately one of four (26.9%; 53/197) disagreed to the statement “My organization provides support (leadership, resources, assistance, etc.) needed to use this guideline”. In statement Q2.25 and Q2.27, the respondents’ perceptions of the guideline’s consistency with available evidence and how clearly the guideline describes this underlying evidence as foundation for the recommendations was explored, and the uncertainty was relatively high for both statements (“Not sure”: 37.2%; 73/196, and 47.2%; 93/197 respectively) (Table 2).

**Table 2:** Response distribution regarding 23 determinants for use of the SNC-16 guideline

**Statement (n)**	**Agree**^a^		**Disagree/Not sure**^b^	
	**n**	**%**	**n**	**%**
***Attitude towards use of the SNC-16 guideline***^a^				
Q2.5 I agree with the content of the SNC-16 guideline (n = 197)	187	**94.9**	10	5.1
Q2.6 Following the guideline will improve care delivery (n = 198)	180	**90.9**	18	9.1
Q2.7 Following the guideline will improve patient outcomes (n = 196)	163	**83.2**	33	16.8
Q2.8 Following the guideline brings advantages to me, my practice or organization, or my patients (n = 198)	181	**91.4**	17	8.6
Q2.9 Following the guideline brings disadvantages to me, my practice or organization, or my patients (n = 197)	25	**12.7**	172	87.3
***Confidence in using the SNC-16 guideline***				
Q2.10 I possess general knowledge about the clinical condition that is needed to use this guideline (n = 198)	191	**96.4**	7	3.6
Q2.11 I was trained in the skills (i.e. technical, procedural, cognitive, etc.) needed to use this guideline (n = 198)	166	**83.8**	32	16.2
Q2.12 I am confident that I possess the skills (i.e. technical, procedural, cognitive, problem-solving, etc.) needed to use this guideline (n = 196)	184	**93.9**	12	6.1
Q2.13 It is among my self-acknowledged professional responsibilities to follow the procedures, actions or activities recommended in this guideline (n = 197)	177	**89.8**	20	10.2
Q2.14 I have the autonomy to make changes needed to follow this guideline (n = 197)	153	**77.7**	44	22.3
***Support from peers and organization in use of the SNC-16 guideline***^a^				
Q2.15 Colleagues in my own organization use the guideline (n = 197)	164	**83.2**	33	16.8
Q2.16 Colleagues outside of my organization use the guideline (n = 196)	60	**30.6**	136	69.4
Q2.17 My organization provides support (leadership, resources, assistance, etc.) needed to use this guideline (n = 197)	120	**60.9**	77	39.1
Q2.18 The procedures, actions or activities recommended in this guideline is easy to incorporate in my practice (n = 193)	184	**95.3**	9	4.7
***Patient and parents’ attitudes towards use of guideline***^a^				
Q2.19 The recommendations in this guideline are consistent with my patients’ values and preferences (n = 197)	139	**70.6**	58	29.4
Q2.20 My patients do, or are likely to accept and follow the recommendations in this guideline (n = 197)	172	**87.3**	25	12.7
***Access and usability of the SNC-16 guideline***^a^				
Q2.21 It is easy to find information in this guideline because the format and layout is easy to navigate (n = 197)	188	**95.4**	9	4.6
Q2.22 The wording of this recommendation is clear and unambiguous (n = 196)	171	**87.2**	25	12.8
Q2.23 The guideline includes or is accompanied by implementation tools (clinician summary, patient summary, algorithm, medical record forms, etc.) (n = 197)	116	**58.9**	81	41.1
Q2.24 Implementation tools included in or with the guideline (clinician summary, patient summary, algorithm, chart forms, etc.) are helpful to me, my practice or organization, or my patients (n = 195)	131	**67.2**	64	32.8
Q2.25 The guideline is consistent with the available evidence (n = 196)	117	**59.7**	79	40.3
Q2.26 The guideline describes whether patient preferences were collected and influenced the guideline questions, methods or recommendations (n = 195)	37	**19.0**	158	81.0
Q2.27 The guideline clearly describes underlying evidence supporting the recommendations (n = 197)	74	**37.6**	123	62.4

^a^Section 2 of CGDQ has 23 items that are subcategorised under five subheadings, as shown in Table 2

^b^Each item is answered on a 1–7 step Likert scale (1 = strongly disagree, 7 = strongly agree). “Not sure” is also a response option. Responses are dichotomized as Disagree/Not Sure (Likert response 1–4 or Not Sure) or Agree (Likert response 5–7 and presented with numbers and percentages. Number of total responses are shown for each statement, as well as percentages for “Agree”, in bold text

### Enablers and barriers

Four types of enablers for guideline use emerged from the compilation of the free-text responses: *ease of use and implementation, alignment with local guidelines and practice, advantages for stakeholders,* and *practicality and accessibility.* Barriers for guideline use were manifested as: *organizational challenges, medical concerns*, and *practical concerns* (Table 3).

**Table 3:** Summarized free-text answers about relevant enablers and barriers for the use of the SNC-16 guideline

**Enablers**
**Ease of use and implementation**
Simple to use
Simple to implement
Clear instructions
Online
Free
Accessibility
**Alignment with local guidelines and practice**
Recommended in local guidelines
Guideline in tune with local practice
Clinical applicability
Generally accepted
**Advantages for stakeholders**
Gives advantages to physicians
Gives advantages to patients
Supports decision making
**Practicality and accessibility**
Format and layout make the guideline easy to use
Available as posters, laminated cards in the emergency room
Takes skull fractures into consideration
Reliable
Known among nurses
Gives support to discharge patients, relieving bed shortages
Included in a collection of validated guidelines online
Patient information included
**Barriers**
**Organizational challenges**
Organizational lack in providing observational units
Organizational lack in guideline endorsement
Limited resources (CT, observational spots, etc.)
Lack of implementational tools
**Medical concerns**
Clinical experience makes the need for the guideline redundant
Fear that the guideline over-triages to CT
GCS not generally used in all hospitals but rather RLS
Inexperience with GCS
Not yet clinically validated
Guidelines can't grasp a complex clinical picture
Crowding of ED making patients/parents uncomfortable with staying for observation
Lack of available evidence
Risk of over-investigation
Worried parents that exaggerate symptoms
Suspected loss of consciousness leading to excessive observation
**Practical concerns**
Lack of time
Other similar guidelines already in use
Availability
Hard to find guideline
Complexity

The free-text responses obtained from questions 3.1 to 3.4 (additional file 2) were independently categorized into types of barriers and enablers by two of the authors (FW, WB) and then compiled in consensus

This section provided participants an opportunity to share thoughts on other determinants that could enable or challenge their use of the guideline. Noteworthy examples of "Enablers" were suggestions to extend the formal implementation among nurses, aiming to achieve a widespread adherence and acceptance of the SNC-16 guideline within all categories of healthcare professionals managing these conditions. Regarding practical concerns, ease of accessibility, e.g. laminated plastic cards in the ED, online versions, simple and unambiguous instructions, were described as enabling use of the guideline. Additionally, the importance of including disseminated guidelines, such as the SNC-16 guideline, into official local guidelines and practices was highlighted. In a broader perspective, a suggestion to gather all relevant guidelines in a bundle of nationally endorsed clinical decision-making tools was also noted.

In contrast, the absence of official organizational endorsement, both on a local and national level, emerged as a potential barrier. A specific concern raised was the fact that many Swedish physicians use the Reaction Level Scale-85 (RLS-85) [27], as opposed to the Glasgow Coma Scale (GCS) [28] recommended in the SNC-16 guideline, for assessment of level of consciousness. This discord was suggested as a barrier to adopting the SNC-16 guideline rising from inexperience in using the GCS. Challenges related to organizational practices, such as the absence of observational units and ED overcrowding, were identified as barriers affecting guideline adherence, possibly instead increasing the use of CT scanning. Within the category of *medical concerns*, participants expressed concern about the risk of over-investigation, encompassing both excessive observation and CT scans, and that the guideline might result in decisions that contradict the clinical judgement of experienced physicians. Concerns about the lack of clinical validation and available evidence were also raised by the respondents. The "practical concerns" category was composed around issues of complexity of guideline, time constraints, and limited availability.

In summary, the free-text responses confirmed already reported key enablers and barriers. They also provided new suggestions regarding the value of interdisciplinary collaboration among healthcare professionals and the importance of organizational structures for guideline adherence.

### Learning style

**Fig. 2:**
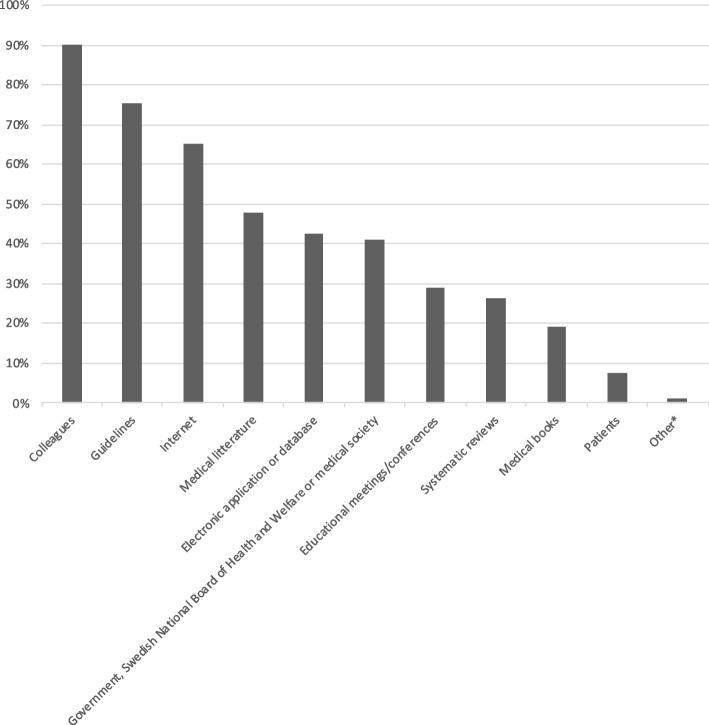
Key sources to guide clinical decision making. 198 respondents provided answers to the multiple-choice question (4.1 in additional file 2) about the usefulness of different sources when seeking support to guide clinical decision-making. *Other = Foamed (free open access medical education) and local guidelines (*n* = 2)

**Fig. 3:**
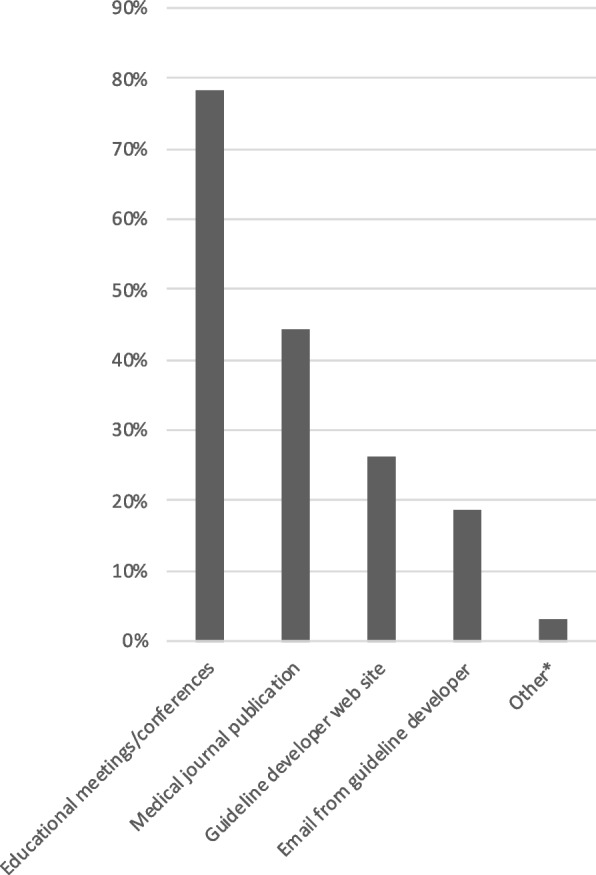
Preferred ways to learn about guidelines. A total of 198 respondents provided answers to this multiple-choice question (4.2 in additional file 2). *Other = Suggested national Swedish collection of guidelines, podcasts, official medical guideline database (“Internetmedicin”), educational lunch sessions, colleagues (*n* = 6)

**Fig. 4:**
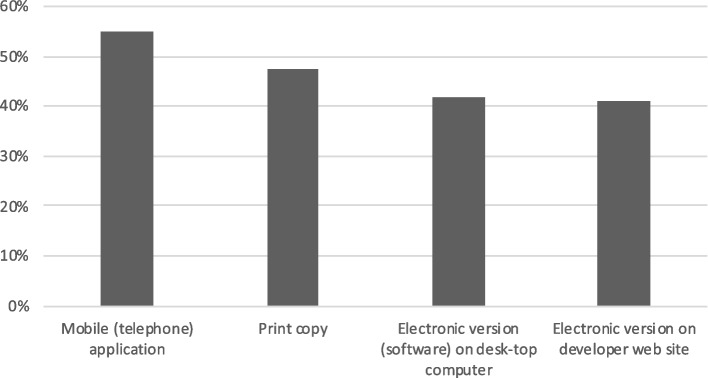
Preferred formats for guidelines, guideline summaries, or guideline tools (*n* = 198, multiple choice)

Most of the respondents reported a preference for consulting colleagues (89.9%; 178/198), guidelines (75.3%; 149/198), and the internet (65.2%; 129/198) to gain knowledge to guide their clinical decisions (Fig. 2). Educational meetings/conferences were the most popular way to learn about guidelines (78.3%; 155/198) (Fig. 3). No clear preference was apparent regarding the optimal format for distribution of guideline material (Fig. 4).

### Associations to demographic variables

Associations between background variables and a subset of determinants were explored in Table 4. There were significant differences between respondents that managed pediatric head injuries regularly, seldom, or rarely in their view of whether following the SNC-16 guideline would improve care delivery (91%; 79/87 versus 94%; 90/96 versus 73%; 11/15) and their view on the support provided from their organization to enable them to use the guideline (73%; 63/86 versus 52%; 50/96 versus 47%; 7/15). Those respondents that believed that guidelines (in general) optimize healthcare delivery and outcomes also had a significantly higher belief in that following the SNC-16 guideline would improve delivered care. There were no significant differences regarding gender, career stage, specialty, size of hospital, location of the respondent’s hospital in Sweden, types of patients managed, or whether the respondent had experience in guideline development for the selected determinants.

**Table 4:** Associations between background factors and selected determinants for guideline use

		**I agree with the content of the SNC-16 guideline**	**Following the guideline will improve care delivery**	**I possess general knowledge about the clinical condition that is needed to use this guideline**	**My organization provides support (leadership, resources, assistance, etc.) needed to use this guideline**	**It is easy to find information in this guideline because the format and layout is easy to navigate**	**The guideline clearly describes underlying evidence supporting the recommendations**
		Agree (n)	Agree (n)	Agree (n)	Agree (n)	Agree (n)	Agree (n)
**Gender**^**a**^	Male	96% (95/99)	89% (89/99)	98% (97/99)	61% (60/99)	96% (95/99)	38% (38/99)
	Female	94% (89/95)	92% (88/96)	95% (91/96)	62% (59/95)	95% (90/95)	35% (35/95)
**Career stage**	Early	96% (27/28)	86% (24/28)	93% (26/28)	64% (18/28)	96% (27/28)	21% (6/28)
	Mid	94% (90/96)	96% (92/96)	98% (94/96)	60% (56/95)	96% (91/95)	39% (37/95)
	Late	96% (70/73)	86% (64/74)	96% (71/74)	62% (46/74)	95% (70/74)	42% (31/74)
**Specialty**	EM	95% (57/60)	84% (53/61)	95% (58/61)	65% (42/61)	91% (55/61)	37% (22/61)
	Surgery	96% (97/101)	93% (94/101)	96% (97/101)	58% (58/101)	97% (98/101)	40% (41/101)
	Other^c^	92% (33/36)	94% (33/36)	100% (36/36)	63% (20/35)	97% (35/35)	34% (11/35)
**Category of hospital**^b^	Large	91% (32/35)	89% (31/35)	100% (35/35)	68% (23/34)	94% (32/34)	38% (13/34)
	Small	96% (155/162)	91% (149/163)	96% (156/163)	60% (97/163)	96% (156/163)	37% (61/163)
**Region of Sweden**	South	95% (137/144)	91% (132/145)	96% (139/145)	62% (89/144)	94% (135/144)	37% (55/144)
	Central	92% (33/36)	89% (32/36)	100% (36/36)	71% (22/36)	100% (36/36)	33% (12/36)
	North	100% (17/17)	94% (16/17)	94% (16/17)	53% (9/17)	100% (17/17)	41% (7/17)
**Types of patients managed**	Children	88% (21/24)	92% (22/24)	100% (24/24)	70% (16/23)	96%(22/23)	44% (10/23)
	Children & adults	96% (165/172)	91% (157/173)	96% (166/173)	60% (103/173)	95% (165/173)	37% (64/173)
**Frequency assessing children with mild head injury**	Regularly	94% (81/86)	91% (79/87)*	98% (85/87)	73% (63/86)*	93% (80/86)	43% (37/86)
	Seldom	96% (92/96)	94% (90/96)*	96% (92/96)	52% (50/96)*	97% (93/96)	33% (32/96)
	Rarely	93% (14/15)	73% (11/15)*	93% (14/15)	47% (7/15)*	100% (15/15)	33% (5/15)
**I have participated in guideline development of one or more guidelines**	Yes	97% (64/66)	92% (61/66)	99% (65/66)	68% (45/66)	94% (62/66)	46% (30/66)
	No	94% (122/130)	90% (118/131)	95% (125/131)	57% (74/130)	96% (125/130)	34% (44/130)
**I believe that guidelines (in general) optimize health care delivery and outcomes**	Yes	95% (178/187)	93% (175/188)*	96% (181/188)	61% (114/188)	95% (179/188)	32% (70/188)
	No^d^	89% (8/9)	44% (4/9)*	100% (9/9)	63% (5/8)	100% (8/8)	38% (3/8)

^*^Chi-square test (or Fisher’s exact test when appropriate) with statistical significance level *p* < 0.05 was used to explore associations. Statistical significance is shown with a*

^a^For gender is one respondent with gender "other" not included in analysis

^b^Hospital size is categorized as small (local and regional) vs large (university and children’s) hospitals

^c^Specialty is reported in three categories (EM = emergency medicine; Surgery = general surgery; other = all other specialties as reported in Table 1, including 2 respondents with both EM and surgery as reported specialty)

^d^For the variable “I believe that guidelines (in general) optimize health care delivery and outcomes”, response options “No” (*n* = 1) and “Unsure” (*n* = 8) have been merged

## Discussion

This cross-sectional survey showed that reported regular use of the passively disseminated SNC-16 guideline for pediatric mTBI was high. The respondents also held a high belief in patient benefit if applying the guideline. Improvements in the reporting of the underlying evidence and appurtenant implementation tools were requested. Barriers, such as lack of organizational support and resources, emerged both in the qualitative and quantitative data. The conveyed perception of determinants for use of the SNC-16 guideline was generally homogenous among the respondents, and independent of varying grouping variables.

The high proportion of regular guideline use (76%) reported in this study is in contrast to other reports, with only 35% adhering to guidelines in a systematic review by Mickan et al. [29] and 43% of prenatal care physicians regularly using a hepatitis C virus screening guideline in a survey by Moore et al. [30]. In a recent report on management routines at an organizational level, 55% of Swedish hospitals based their local recommendation in part or fully on the SNC-16 guideline [13]. The reason for this seemingly successful non-facilitated dissemination of the SNC-16 guideline in Sweden is unclear, although some plausible causes can be hypothesized. There is a lack of alternative, validated guidelines in Scandinavia. Also, the guidelines were published in the most common national journal and on the most commonly used web tool for doctors [26, 31]. Additionally, a recent, non-intervention multi-center study, validated a set of pediatric mTBI guidelines in the Scandinavian healthcare system [32].

Pathman et al. [33] developed a four-step model for “leakage” of guideline evidence, from awareness to final adherence, outlining the concept of progressive loss of research evidence from guideline publication to clinical practice. The drop-off, or “leakage”, in each step of the Pathman model was estimated to be 15% in the systematic review by Mickan et al. [29]. The first step, *awareness* of the SNC-16 guideline, is not explicitly measured in the CGDQ. The second step is *agreement* with the content. If assuming that “regular use” corresponds to *adoption* or *adherence* in the Pathman framework, the leakage in this study would be between 9.25% (*agreement* to *adoption* to *adherence*) and 18.5% (*agreement* to *adoption*). This may raise attention to a possible, although not ascertained, discrepancy worth some effort to address in future updates of the guideline, also when considering the design of an implementation strategy. There was, for example, an uncertainty among our respondents concerning the guideline’s consistency with available evidence, which may act as a barrier for adoption and adherence. The guideline format and layout were acknowledged as easy to navigate, with clear and unambiguous wording, which may on the other hand facilitate adoption and adherence and efforts to preserve it may be beneficial [17].

In pediatric guidelines for mTBI, there has been a successive development from dichotomous prediction models based on single assessments [34, 35], to risk group stratification at several levels (three to five) at one single time-point [5, 14], and more recently to multiple risk groups and assessments at several time-points under observation in ED [36]. Whether the ambition to increase diagnostic accuracy via increasingly complicated flow chart structures will, at some point, limit the accessibility, final adoption and adherence to a guideline remains to be investigated, even though there have been dedicated efforts to investigate optimal implementation pathways and implementation outcome for newer mTBI guidelines both in Australia/New Zealand [36, 37] and the US [38–42]. Among the Swedish respondents, a high belief in the benefit for the patients of using the SNC-16 guideline was reported in this study, which may imply that the basic flowchart structure of the clinical decision rule that is central to the guideline is feasible for the Scandinavian setting. A recent systematic review of trends in guideline implementation showed that even if more studies investigate and tailor interventions to facilitate implementation of a guideline, with most studies reporting effect, studies that did not plan specific implementation measures also achieved impact [20]. Causes for a seemingly successful dissemination of the SNC-16 guideline could therefore be numerous.

Potential barriers for implementation of the SNC-16 guideline could be identified within different types of determinants. Over one quarter of our respondents stated a lack of organizational support needed to use the guideline. Organizational barriers affect uptake of recommendations and a top-down drive of change from medical managers is likely important for adoption of a guideline, identifying team and organization leaders as a target for interventions in future implementation planning [39, 43]. Lack of resources (e.g., observational units, CT accessibility) also seems to pose an organizational challenge in Swedish health care.

Another relevant issue are the implementation tools accompanying the SNC-16 guideline. Respondents were unsure about which tools are included in the guideline and the helpfulness of these tools. This uncertainty was also expressed as a barrier in the free-text answers. Many respondents seem to prefer electronic tools and further improvements may include development of electronic educational tools/websites and integration with electronic health record-based systems, an aspect that has been identified in other populations [37–39, 41]. The need for developing more concise implementation tools, both digital and in print, was identified in an interview study investigating experience and use of the CDC pediatric mTBI guidelines in rural areas in US [38]. Recently, an evaluation of a generic model to integrate decision aids for shared decision making into electronic evidence summaries with adjacent guidelines showed promising results and may be applicable also for pediatric TBI in the future [44]. Another area amenable to improvements is the description of the underlying evidence supporting the recommendations, where only 37.6% agreed that the description was clear. This finding is in contrast to a survey by Sawka et al. [45], also using the CGDQ, which showed that 92.3% agreed that the evidence underlying the evaluated US thyroid guideline was clearly described.

More than half of the respondents sought guidance for their clinical decision-making from colleagues (90%), guidelines (75%), or the internet (65%) and preferred to learn about guidelines via educational meetings and conferences (78%). Sawka et al. [45], reported somewhat different results regarding the thyroid guideline, where the most common sources for knowledge were medical literature (88.1%), guidelines (87.2%), and colleagues (65.6%). The reported need for discussion with colleagues and learning via meetings/conferences may underscore the need for understanding stakeholders’ views of how to manage mTBI in children. Many respondents were unsure about practice in other settings, and educational meetings may fill an important knowledge gap in this respect. Daugherty et al. [38], who evaluated the implementation of the CDC pediatric mTBI guideline in a rural area in the US, identified a perceived lack of access to mTBI specialists and discussed the telemonitoring ECHO model as an example where health care providers could meet in a virtual community and discuss cases. There are reports on the application of this model in pediatric emergency care and pediatric mTBI [46, 47]. In a recent systematic review, education of professionals was a commonly utilized intervention in guideline implementation planning [20]. Another review by Chan et al. [48] reported a positive impact through specific interventions, namely educational outreach, audit, and feedback. There was a significant association between familiarity with assessing pediatric mTBI and the perceived benefit of adherence to the recommendations. This association might be explained by senior physicians managing this condition more seldom, and when doing so relying on their clinical judgement and solid experience rather than a clinical practice guideline [37].

There are several limitations to consider when interpreting the results from this survey. The low total response rate of 43.4% (analyzable response rate 33.3%) implies a potential responder bias. The high reported use of the guideline could be an effect of sampling bias due to the modified snowball sampling method, for example if the respondents more commonly recommended colleagues with similar education, value base, or within the same organization. Nevertheless, our sampling strategy and different e-mail address collection strategies offered a good opportunity to maximize and optimize respondent relevance by drawing on snowball sampling, the ED physician community, and the ongoing guideline implementation. The background information does not, however, indicate a widespread bias among respondents as the distribution of gender, career stage, category of hospital, part of Sweden, and types of patients managed is reasonable from a Swedish healthcare perspective. Another risk worth mentioning is that of contamination, in the form of an observer effect. There has been an intense focus in Sweden on pediatric mTBI management as an effect of the ongoing guideline validation efforts. The validation study [32] is strictly observational but has inevitably set focus on the SNC-16 guidelines and the investigators behind these. However, the use of an e-mail recipient list from the 2022 study [13] is unlikely to have contaminated the responses as there was only one respondent from each of the 66 hospitals in that study. Another limitation is the cross-sectional design, addressing the physicians’ perceptions of their own actions, leaving room for deviation from the reported views in actual patient management decisions.

## Conclusions

This cross-sectional survey on determinants for use of the Scandinavian guideline for management of mild and moderate head injury in children suggests that use of the guideline is high in our sample of ED providers in Sweden. In updated versions of the guideline, focus on improving implementation tools and descriptions of the underlying evidence may further facilitate adoption and adherence. Measures to improve organizational support for guideline use and involvement of patient representatives should also be considered.

### Supplementary Information

Additional file 1. STROBE statement.

Additional file 2. Survey.

Additional file 3. Unmerged response rates.

## Data Availability

Pseudonymized datasets used and analyzed during the current study are available from the corresponding author on reasonable request.

## Abbreviations

SNC: Scandinavian Neurotrauma Committee
CT: Computed tomography
ED: Emergency department
CGDQ: Clinical Guideline Determinants Questionnaire
mTBI: Mild traumatic brain injury
GCS: Glasgow Coma Scale
RLS-85: Reaction Level Scale -85
CDC: Centers for Disease Control
