# Supplementary material for: Pediatric head injury guideline use in Sweden: a cross-sectional survey on determinants for successful implementation of a clinical practice guideline
Source: BMC Health Serv Res. 2024 Aug 21;24:965. doi: 10.1186/s12913-024-11423-z (PMC11340051; doi:10.1186/s12913-024-11423-z)
Supplement: Supplementary file 2 — Additional file 2. Survey. [file 12913_2024_11423_MOESM2_ESM.docx]

Additional file 2 - Survey

Scandinavian Neurotrauma Committee

presents

*"Factors influencing implementation and guideline adherence"*

**A nationalwide cross-sectional survey**

**Thank you for your participation!**

**Regarding children with head trauma...**

| 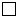 | I regularly manage children with head trauma in the emergency department. |  |  |
| --- | --- | --- | --- |
| 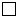 | I do not regularly manage these patients but I know colleagues in my hospital that does. |  |  |
| 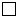 | I do not manage these patients and I do not know any colleagues in my hospital that does. |  |  |

**Click "Next" at the bottom of this page.**

1. Background information

 Any questions? Contact: info@shipp.se

**1.1 Name of your hospital:**

| 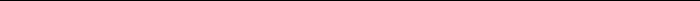 |
| --- |

**1.2 Gender:**

| 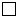 | Male |  |  |
| --- | --- | --- | --- |
| 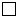 | Female |  |  |
| 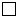 | Prefer not to respond |  |  |

**1.3 Career stage:**

| 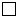 | Early career (Intern, underläkare/AT-läkare) |  |  |
| --- | --- | --- | --- |
| 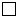 | Mid-career (Residency, ST-läkare) |  |  |
| 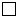 | Late career (Consultant, specialist) |  |  |

**1.4 In what field/specialty are you currently working?**

| 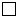 | Pediatric medicine |  |  |
| --- | --- | --- | --- |
| 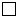 | Surgery |  |  |
| 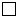 | Pediatric surgery |  |  |
| 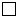 | Emergency medicine |  |  |
| 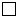 | Neurology |  |  |
| 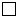 | Pediatric neurology |  |  |
| 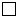 | Neurosurgery |  |  |
| 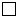 | Internal medicine |  |  |
| 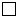 | Orthopedics |  |  |
| 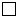 | Pediatric orthopedics |  |  |
| 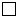 | Other | | |
|  | 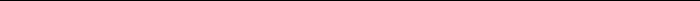 | | |

**1.5 I believe that guidelines (in general) optimize health care delivery and outcomes by supporting patient-clinician communication and decision-making**

| 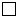 | Yes |  |  |
| --- | --- | --- | --- |
| 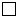 | No |  |  |
| 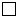 | Unsure |  |  |

**1.6 I have participated in the development of one or more guidelines**

| 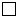 | Yes |  |  |
| --- | --- | --- | --- |
| 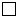 | No |  |  |

**1.7 What category/-es of patients with head injury do you treat and assess at your emergency department?**

| 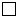 | Children only |  |  |
| --- | --- | --- | --- |
| 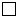 | Adults only |  |  |
| 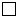 | Both children and adults |  |  |

**The SNC-16 guideline**

 In 2016, SNC (Scandinavian Neurotrauma Committee) published management guidelines of children with minimal, mild and moderate headtrauma.

 
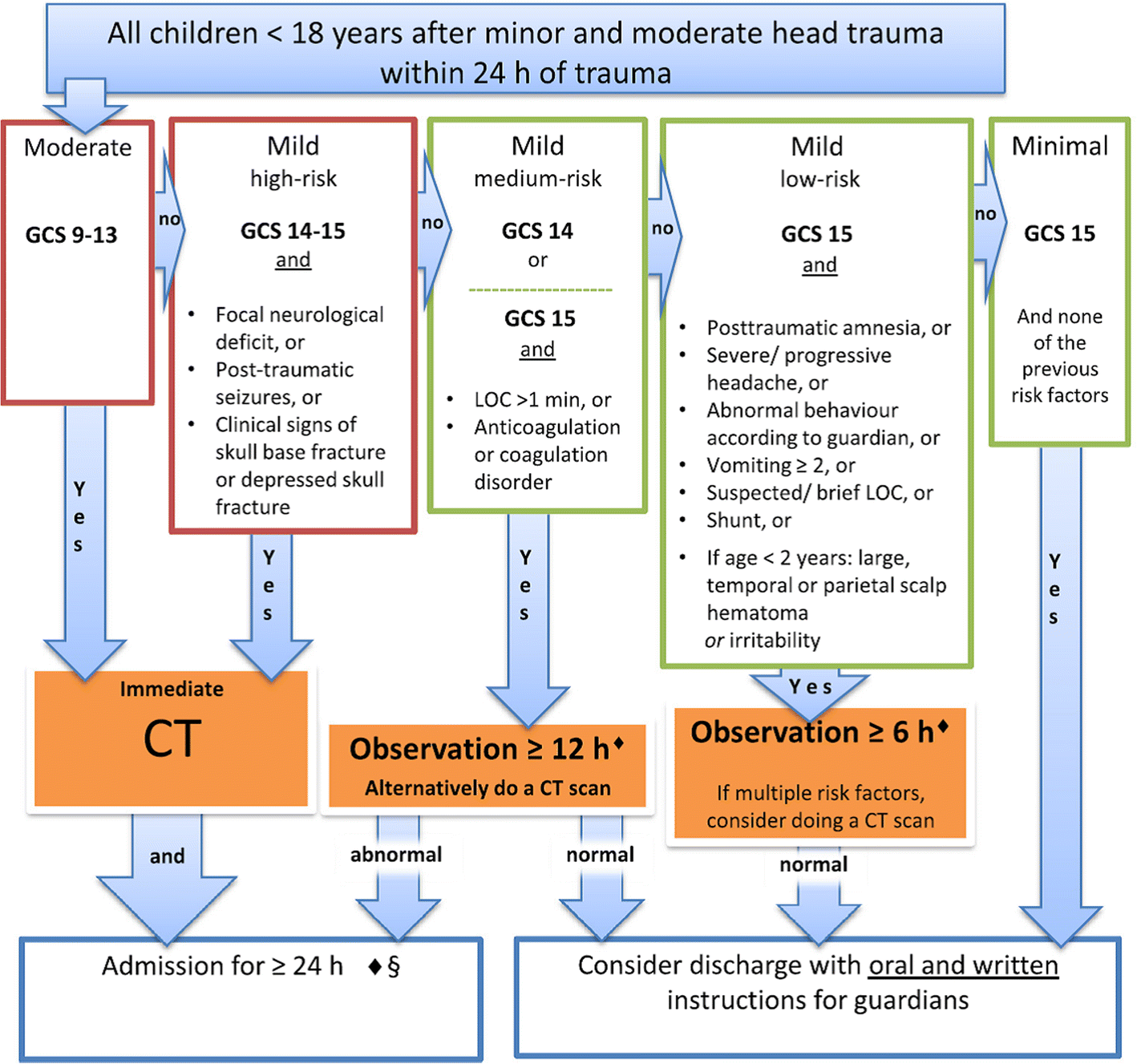


*Astrand R, Rosenlund C, Unden J, Scandinavian Neurotrauma Committee (SNC). Scandinavian guidelines for initial management of minor and moderate head trauma in children. BMC Med 2016;14:33–016-0574*

Further information:

[Läkartidningen](http://www.lakartidningen.se/Klinik-och-vetenskap/Klinisk-oversikt/2017/04/Nya-skandinaviska-riktlinjer-for-att-handlagga-skallskador-hos-barn/)

[PubMed](https://www.ncbi.nlm.nih.gov/pmc/articles/PMC4758024/)

Any questions? Contact: info@shipp.se

**2. Determinants of guideline use of the SNC-guidelines -16 (pediatric version)**

 Any questions? Contact: info@shipp.se

**2.1 How often do you see pediatric patients with mild head injury?**

| 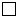 | Daily |  |  |
| --- | --- | --- | --- |
| 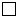 | Several times a week |  |  |
| 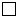 | 1-3 times/month |  |  |
| 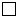 | 5-10 times/year |  |  |
| 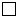 | 1-4 times/year |  |  |
| 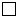 | Less then once a year |  |  |

**2.2 What is your level of awareness of/familiarity with the SNC-16 guideline?  
 
Choose the response that best matches your scenario**

| 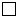 | I was not aware prior to this questionnaire |  |  |
| --- | --- | --- | --- |
| 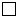 | I am aware of the guideline but have not read it |  |  |
| 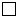 | I have read all or some of the guideline on one occasion then never again |  |  |
| 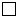 | I have read all or some of the guideline on multiple occasions |  |  |
| 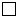 | Other (specify): | | |
|  | 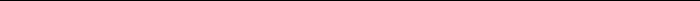 | | |

**2.3 What is your intended or actual use of the SNC-16 guideline?  
 
Choose the response that best matches your scenario**

| 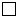 | I have never used the guideline and do not plan to |  |  |
| --- | --- | --- | --- |
| 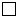 | I have never used the guideline but will consider using it |  |  |
| 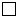 | I have never used the guideline but will use it |  |  |
| 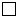 | I have used the guideline once only |  |  |
| 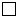 | I have used the guideline a few times |  |  |
| 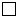 | I regularly use the guideline |  |  |
| 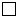 | Other (specify): | | |
|  | 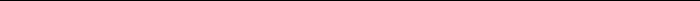 | | |

**2.4 Others expect me to use the procedures, actions or activities recommended in this guideline  
 
*Choose all that apply***

| 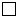 | Patients |  |  |
| --- | --- | --- | --- |
| 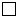 | Colleagues |  |  |
| 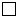 | Managers or executives in my organization |  |  |
| 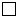 | Monitoring agency (Socialstyrelsen) |  |  |
| 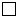 | Government |  |  |
| 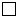 | Professional Society |  |  |
| 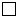 | Other (specify): | | |
|  | 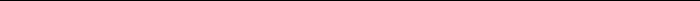 | | |

**Attitude towards use of the SNC-16 guideline  
 
To what extent do you agree with the following statements  
 
1: Strongly disagree  
7: Strongly agree**

|  | 1 - Strongly disagree | 2 | 3 | 4 | 5 | 6 | 7 - Strongly agree | Not sure |
| --- | --- | --- | --- | --- | --- | --- | --- | --- |
| 2.5 I agree with the content of the SNC-16 guideline | 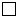 | 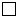 | 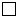 | 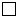 | 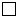 | 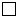 | 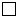 | 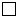 |
| 2.6 Following the guideline will improve care delivery | 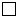 | 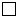 | 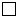 | 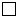 | 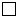 | 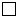 | 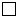 | 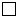 |
| 2.7 Following the guideline will improve patient outcomes | 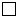 | 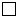 | 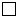 | 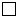 | 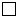 | 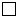 | 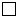 | 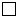 |
| 2.8 Following the guideline brings advantages to me, my practice or organization, or my patients (i.e. supports communication and decision-making, etc.) | 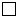 | 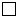 | 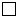 | 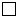 | 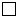 | 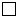 | 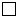 | 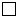 |
| 2.9 Following the guideline brings disadvantages to me, my practice or organization, or my patients (i.e. time, costs, etc.) | 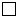 | 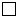 | 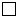 | 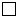 | 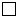 | 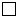 | 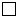 | 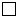 |

**Confidence in using the SNC-16 guideline  
 
To what extent do you agree with the following statements  
 
1: Strongly disagree  
7: Strongly agree**

|  | 1 - Strongly disagree | 2 | 3 | 4 | 5 | 6 | 7 - Strongly agree | Not sure |
| --- | --- | --- | --- | --- | --- | --- | --- | --- |
| 2.10 I possess general knowledge about the clinical condition that is needed to use this guideline | 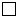 |  |  |  |  |  |  |  |
| 2.11 I was trained in the skills (i.e. technical, procedural, cognitive, etc.) needed to use this guideline |  |  |  |  |  |  |  |  |
| 2.12 I am confident that I possess the skills (i.e. technical, procedural, cognitive, problem-solving, etc.) needed to use this guideline |  |  |  |  |  |  |  |  |
| 2.13 It is among my self-acknowledged professional responsibilities to follow the procedures, actions or activities recommended in this guideline |  |  |  |  |  |  |  |  |
| 2.14 I have the autonomy to make changes needed to follow this this guideline |  |  |  |  |  |  |  |  |

**Support from peers and organization in use of the SNC-16 guideline  
 
To what extent do you agree with the following statements  
 
1: Strongly disagree  
7: Strongly agree**

|  | 1 - Strongly disagree | 2 | 3 | 4 | 5 | 6 | 7 - Strongly agree | Not sure |
| --- | --- | --- | --- | --- | --- | --- | --- | --- |
| 2.15 Colleagues in my own organization use the guideline |  |  |  |  |  |  |  |  |
| 2.16 Colleagues outside of my organization use the guideline |  |  |  |  |  |  |  |  |
| 2.17 My organization provides support (leadership, resources, assistance, etc.) needed to use this guideline |  |  |  |  |  |  |  |  |
| 2.18 The procedures, actions or activities recommended in this guideline is easy to incorporate in my practice |  |  |  |  |  |  |  |  |

**Patient and parents attitudes towards use of guideline  
 
To what extent do you agree with the following statements  
 
1: Strongly disagree  
7: Strongly agree**

|  | 1 - Strongly disagree | 2 | 3 | 4 | 5 | 6 | 7 - Strongly agree | Not sure |
| --- | --- | --- | --- | --- | --- | --- | --- | --- |
| 2.19 The recommendations in this guideline are consistent with my patients’ values and preferences |  |  |  |  |  |  |  |  |
| 2.20 My patients do, or are likely to accept and follow the recommendations in this guideline |  |  |  |  |  |  |  |  |

**Access and usability of the SNC-16 guideline  
 
To what extent do you agree with the following statements  
 
1: Strongly disagree  
7: Strongly agree**

|  | 1 - Strongly disagree | 2 | 3 | 4 | 5 | 6 | 7 - Strongly agree | Not sure |
| --- | --- | --- | --- | --- | --- | --- | --- | --- |
| 2.21 It is easy to find information in this guideline because the format and layout is easy to navigate |  |  |  |  |  |  |  |  |
| 2.22 The wording of this recommendation is clear and unambiguous |  |  |  |  |  |  |  |  |
| 2.23 The guideline includes or is accompanied by implementation tools (clinician summary, patient summary, algorithm, medical record forms, etc.) |  |  |  |  |  |  |  |  |
| 2.24 Implementation tools included in or with the guideline (clinician summary, patient summary, algorithm, chart forms, etc.) are helpful to me, my practice or organization, or my patients |  |  |  |  |  |  |  |  |
| 2.25 The guideline is consistent with the available evidence |  |  |  |  |  |  |  |  |
| 2.26 The guideline describes whether patient preferences were collected and influenced the guideline questions, methods or recommendations |  |  |  |  |  |  |  |  |
| 2.27 The guideline clearly describes underlying evidence   supporting the recommendations |  |  |  |  |  |  |  |  |

**3. Other determinants**

Any questions? Contact: info@shipp.se

**Enablers**

**3.1 What is the single most important factor noted above that does/will enable your use of this guideline?**

|  |
| --- |

**3.2 What is the single most important factor NOT noted above that does/will enable your use of this guideline?**

|  |
| --- |

**Barriers**

**3.3 What is the single most important factor noted above that does/will challenge your use of this guideline?**

|  |
| --- |

**3.4 What is the single most important factor NOT noted above that does/will challenge your use of this guideline?**

|  |
| --- |

**4. Learning style**

Any questions? Contact: info@shipp.se

**4.1 What sources do you most often consult for knowledge to guide clinical decision making?  
 
Choose all that apply**

|  | Colleagues |  |  |
| --- | --- | --- | --- |
|  | Patients |  |  |
|  | Medical literature |  |  |
|  | Electronic application or database |  |  |
|  | Internet |  |  |
|  | Guidance from government, regulatory agency (socialstyrelsen) or medical society |  |  |
|  | Educational meetings/conferences |  |  |
|  | Medical books |  |  |
|  | Systematic reviews |  |  |
|  | Guidelines |  |  |
|  | Other (specify): | | |
|  |  | | |

**4.2 How do you prefer to learn about guidelines?  
 
 
Choose all that apply**

|  | Educational meetings/conferences |  |  |
| --- | --- | --- | --- |
|  | Guideline developer web site |  |  |
|  | Email from guideline developer |  |  |
|  | Medical journal publication |  |  |
|  | Other (specify): | | |
|  |  | | |

**4.3 What is your preferred format for guidelines, guideline summaries or guideline tools?   
 
Choose all that apply**

|  | Mobile (telephone) application |  |  |
| --- | --- | --- | --- |
|  | Electronic version (software) on desk-top computer |  |  |
|  | Electronic version on developer web site |  |  |
|  | Print copy |  |  |
|  | Other (specify): | | |
|  |  | | |

**4.4 Did you understand the language of the present survey?**

|  | Yes |  |  |
| --- | --- | --- | --- |
|  | No (please comment below) | | |
|  |  | | |
|  |  | | |

**5.1 We kindly ask you to list 5-10 of your colleagues who also work with management of paediatric head trauma in the emergency setting. It could be colleagues from both your hospital or from other Swedish hospitals. We will use the adresses to send this questionnaire and ask them to respond.**

| Colleague 1- email |
| --- |
|  |
| Colleague 2- email |
|  |
| Colleague 3- email |
|  |
| Colleague 4- email |
|  |
| Colleague 5- email |
|  |
| Colleague 6- email |
|  |
| Colleague 7- email |
|  |
| Colleague 8- email |
|  |
| Colleague 9- email |
|  |
| Colleague 10- email |
|  |
