# Supplementary material for: Pediatric head injury guideline use in Sweden: a cross-sectional survey on determinants for successful implementation of a clinical practice guideline
Source: BMC Health Serv Res. 2024 Aug 21;24:965. doi: 10.1186/s12913-024-11423-z (PMC11340051; doi:10.1186/s12913-024-11423-z)
Supplement: Supplementary file 3 — Additional file 3. Unmerged response rates. [file 12913_2024_11423_MOESM3_ESM.docx]

Additional file 3. Unmerged response rates

For the manuscript:

*Pediatric head injury guideline use in Sweden: a cross-sectional survey on determinants for successful implementation of a clinical practice guideline.*

| **Q 2.3 What is your intended or actual use of the SNC-16 guideline?** | | | | | |
| --- | --- | --- | --- | --- | --- |
|  | | Frequency | Percent | Valid Percent | Cumulative Percent |
| Valid | I have never used the guideline but will consider using it | 12 | 6,1 | 6,2 | 6,2 |
|  | I have never used the guideline but will use it | 1 | ,5 | ,5 | 6,7 |
|  | I have used the guideline once only | 2 | 1,0 | 1,0 | 7,7 |
|  | I have used the guideline a few times | 31 | 15,7 | 15,9 | 23,6 |
|  | I regularly use the guideline | 149 | 75,3 | 76,4 | 100,0 |
|  | Total | 195 | 98,5 | 100,0 |  |
| Missing | System | 3 | 1,5 |  |  |
| Total | | 198 | 100,0 |  |  |

| **Q 2.5 I agree with the content of the SNC-16 guideline** | | | | | |
| --- | --- | --- | --- | --- | --- |
|  | | Frequency | Percent | Valid Percent | Cumulative Percent |
| Valid | 4 | 4 | 2,0 | 2,0 | 2,0 |
|  | 5 | 31 | 15,7 | 15,7 | 17,8 |
|  | 6 | 77 | 38,9 | 39,1 | 56,9 |
|  | 7 | 79 | 39,9 | 40,1 | 97,0 |
|  | Not sure | 6 | 3,0 | 3,0 | 100,0 |
|  | Total | 197 | 99,5 | 100,0 |  |
| Missing | System | 1 | ,5 |  |  |
| Total | | 198 | 100,0 |  |  |

| **Q 2.6 Following the guideline will improve care delivery** | | | | | |
| --- | --- | --- | --- | --- | --- |
|  | | Frequency | Percent | Valid Percent | Cumulative Percent |
| Valid | 2 | 2 | 1,0 | 1,0 | 1,0 |
|  | 3 | 3 | 1,5 | 1,5 | 2,5 |
|  | 4 | 6 | 3,0 | 3,0 | 5,6 |
|  | 5 | 31 | 15,7 | 15,7 | 21,2 |
|  | 6 | 78 | 39,4 | 39,4 | 60,6 |
|  | 7 | 71 | 35,9 | 35,9 | 96,5 |
|  | Not sure | 7 | 3,5 | 3,5 | 100,0 |
|  | Total | 198 | 100,0 | 100,0 |  |

| **Q 2.7 Following the guideline will improve patient outcomes** | | | | | |
| --- | --- | --- | --- | --- | --- |
|  | | Frequency | Percent | Valid Percent | Cumulative Percent |
| Valid | 1 | 1 | ,5 | ,5 | ,5 |
|  | 2 | 1 | ,5 | ,5 | 1,0 |
|  | 3 | 4 | 2,0 | 2,0 | 3,1 |
|  | 4 | 4 | 2,0 | 2,0 | 5,1 |
|  | 5 | 43 | 21,7 | 21,9 | 27,0 |
|  | 6 | 68 | 34,3 | 34,7 | 61,7 |
|  | 7 | 52 | 26,3 | 26,5 | 88,3 |
|  | Not sure | 23 | 11,6 | 11,7 | 100,0 |
|  | Total | 196 | 99,0 | 100,0 |  |
| Missing | System | 2 | 1,0 |  |  |
| Total | | 198 | 100,0 |  |  |

| **Q 2.8 Following the guideline brings advantages to me, my practice or organization, or my patients (i.e. supports communication and decision-making, etc.)** | | | | | |
| --- | --- | --- | --- | --- | --- |
|  | | Frequency | Percent | Valid Percent | Cumulative Percent |
| Valid | 1 | 1 | ,5 | ,5 | ,5 |
|  | 3 | 4 | 2,0 | 2,0 | 2,5 |
|  | 4 | 4 | 2,0 | 2,0 | 4,5 |
|  | 5 | 31 | 15,7 | 15,7 | 20,2 |
|  | 6 | 69 | 34,8 | 34,8 | 55,1 |
|  | 7 | 81 | 40,9 | 40,9 | 96,0 |
|  | Not sure | 8 | 4,0 | 4,0 | 100,0 |
|  | Total | 198 | 100,0 | 100,0 |  |

| **Q 2.9 Following the guideline brings disadvantages to me, my practice or organization, or my patients (i.e. time, costs, etc.)** | | | | | |
| --- | --- | --- | --- | --- | --- |
|  | | Frequency | Percent | Valid Percent | Cumulative Percent |
| Valid | 1 | 67 | 33,8 | 34,0 | 34,0 |
|  | 2 | 81 | 40,9 | 41,1 | 75,1 |
|  | 3 | 12 | 6,1 | 6,1 | 81,2 |
|  | 4 | 7 | 3,5 | 3,6 | 84,8 |
|  | 5 | 10 | 5,1 | 5,1 | 89,8 |
|  | 6 | 7 | 3,5 | 3,6 | 93,4 |
|  | 7 | 8 | 4,0 | 4,1 | 97,5 |
|  | Not sure | 5 | 2,5 | 2,5 | 100,0 |
|  | Total | 197 | 99,5 | 100,0 |  |
| Missing | System | 1 | ,5 |  |  |
| Total | | 198 | 100,0 |  |  |

| **Q 2.10 I possess general knowledge about the clinical condition that is needed to use this guideline** | | | | | |
| --- | --- | --- | --- | --- | --- |
|  | | Frequency | Percent | Valid Percent | Cumulative Percent |
| Valid | 4 | 4 | 2,0 | 2,0 | 2,0 |
|  | 5 | 35 | 17,7 | 17,7 | 19,7 |
|  | 6 | 73 | 36,9 | 36,9 | 56,6 |
|  | 7 | 83 | 41,9 | 41,9 | 98,5 |
|  | Not sure | 3 | 1,5 | 1,5 | 100,0 |
|  | Total | 198 | 100,0 | 100,0 |  |

| **Q 2.11 I was trained in the skills (i.e. technical, procedural, cognitive, etc.) needed to use this guideline** | | | | | |
| --- | --- | --- | --- | --- | --- |
|  | | Frequency | Percent | Valid Percent | Cumulative Percent |
| Valid | 1 | 3 | 1,5 | 1,5 | 1,5 |
|  | 2 | 7 | 3,5 | 3,5 | 5,1 |
|  | 3 | 7 | 3,5 | 3,5 | 8,6 |
|  | 4 | 13 | 6,6 | 6,6 | 15,2 |
|  | 5 | 34 | 17,2 | 17,2 | 32,3 |
|  | 6 | 59 | 29,8 | 29,8 | 62,1 |
|  | 7 | 73 | 36,9 | 36,9 | 99,0 |
|  | Not sure | 2 | 1,0 | 1,0 | 100,0 |
|  | Total | 198 | 100,0 | 100,0 |  |

| **Q 2.12 I am confident that I possess the skills (i.e. technical, procedural, cognitive, problem-solving, etc.) needed to use this guideline** | | | | | |
| --- | --- | --- | --- | --- | --- |
|  | | Frequency | Percent | Valid Percent | Cumulative Percent |
| Valid | 2 | 2 | 1,0 | 1,0 | 1,0 |
|  | 3 | 2 | 1,0 | 1,0 | 2,0 |
|  | 4 | 6 | 3,0 | 3,1 | 5,1 |
|  | 5 | 17 | 8,6 | 8,7 | 13,8 |
|  | 6 | 76 | 38,4 | 38,8 | 52,6 |
|  | 7 | 91 | 46,0 | 46,4 | 99,0 |
|  | Not sure | 2 | 1,0 | 1,0 | 100,0 |
|  | Total | 196 | 99,0 | 100,0 |  |
| Missing | System | 2 | 1,0 |  |  |
| Total | | 198 | 100,0 |  |  |

| **Q 2.13 It is among my self-acknowledged professional responsibilities to follow the procedures, actions or activities recommended in this guideline** | | | | | |
| --- | --- | --- | --- | --- | --- |
|  | | Frequency | Percent | Valid Percent | Cumulative Percent |
| Valid | 1 | 1 | ,5 | ,5 | ,5 |
|  | 2 | 1 | ,5 | ,5 | 1,0 |
|  | 3 | 1 | ,5 | ,5 | 1,5 |
|  | 4 | 11 | 5,6 | 5,6 | 7,1 |
|  | 5 | 23 | 11,6 | 11,7 | 18,8 |
|  | 6 | 78 | 39,4 | 39,6 | 58,4 |
|  | 7 | 76 | 38,4 | 38,6 | 97,0 |
|  | Not sure | 6 | 3,0 | 3,0 | 100,0 |
|  | Total | 197 | 99,5 | 100,0 |  |
| Missing | System | 1 | ,5 |  |  |
| Total | | 198 | 100,0 |  |  |

| **Q 2.14 I have the autonomy to make changes needed to follow this this guideline** | | | | | |
| --- | --- | --- | --- | --- | --- |
|  | | Frequency | Percent | Valid Percent | Cumulative Percent |
| Valid | 1 | 3 | 1,5 | 1,5 | 1,5 |
|  | 2 | 5 | 2,5 | 2,5 | 4,1 |
|  | 3 | 5 | 2,5 | 2,5 | 6,6 |
|  | 4 | 10 | 5,1 | 5,1 | 11,7 |
|  | 5 | 23 | 11,6 | 11,7 | 23,4 |
|  | 6 | 66 | 33,3 | 33,5 | 56,9 |
|  | 7 | 64 | 32,3 | 32,5 | 89,3 |
|  | Not sure | 21 | 10,6 | 10,7 | 100,0 |
|  | Total | 197 | 99,5 | 100,0 |  |
| Missing | System | 1 | ,5 |  |  |
| Total | | 198 | 100,0 |  |  |

| **Q 2.15 Colleagues in my own organization use the guideline** | | | | | |
| --- | --- | --- | --- | --- | --- |
|  | | Frequency | Percent | Valid Percent | Cumulative Percent |
| Valid | 1 | 1 | ,5 | ,5 | ,5 |
|  | 2 | 1 | ,5 | ,5 | 1,0 |
|  | 3 | 2 | 1,0 | 1,0 | 2,0 |
|  | 4 | 10 | 5,1 | 5,1 | 7,1 |
|  | 5 | 33 | 16,7 | 16,8 | 23,9 |
|  | 6 | 71 | 35,9 | 36,0 | 59,9 |
|  | 7 | 60 | 30,3 | 30,5 | 90,4 |
|  | Not sure | 19 | 9,6 | 9,6 | 100,0 |
|  | Total | 197 | 99,5 | 100,0 |  |
| Missing | System | 1 | ,5 |  |  |
| Total | | 198 | 100,0 |  |  |

| **Q 2.16 Colleagues outside of my organization use the guideline** | | | | | |
| --- | --- | --- | --- | --- | --- |
|  | | Frequency | Percent | Valid Percent | Cumulative Percent |
| Valid | 2 | 2 | 1,0 | 1,0 | 1,0 |
|  | 3 | 3 | 1,5 | 1,5 | 2,6 |
|  | 4 | 11 | 5,6 | 5,6 | 8,2 |
|  | 5 | 19 | 9,6 | 9,7 | 17,9 |
|  | 6 | 26 | 13,1 | 13,3 | 31,1 |
|  | 7 | 15 | 7,6 | 7,7 | 38,8 |
|  | Not sure | 120 | 60,6 | 61,2 | 100,0 |
|  | Total | 196 | 99,0 | 100,0 |  |
| Missing | System | 2 | 1,0 |  |  |
| Total | | 198 | 100,0 |  |  |

| **Q 2.17 My organization provides support (leadership, resources, assistance, etc.) needed to use this guideline** | | | | | |
| --- | --- | --- | --- | --- | --- |
|  | | Frequency | Percent | Valid Percent | Cumulative Percent |
| Valid | 1 | 4 | 2,0 | 2,0 | 2,0 |
|  | 2 | 8 | 4,0 | 4,1 | 6,1 |
|  | 3 | 17 | 8,6 | 8,6 | 14,7 |
|  | 4 | 24 | 12,1 | 12,2 | 26,9 |
|  | 5 | 30 | 15,2 | 15,2 | 42,1 |
|  | 6 | 50 | 25,3 | 25,4 | 67,5 |
|  | 7 | 40 | 20,2 | 20,3 | 87,8 |
|  | Not sure | 24 | 12,1 | 12,2 | 100,0 |
|  | Total | 197 | 99,5 | 100,0 |  |
| Missing | System | 1 | ,5 |  |  |
| Total | | 198 | 100,0 |  |  |

| **Q 2.18 The procedures, actions or activities recommended in this guideline is easy to incorporate in my practice** | | | | | |
| --- | --- | --- | --- | --- | --- |
|  | | Frequency | Percent | Valid Percent | Cumulative Percent |
| Valid | 2 | 2 | 1,0 | 1,0 | 1,0 |
|  | 4 | 6 | 3,0 | 3,1 | 4,1 |
|  | 5 | 28 | 14,1 | 14,5 | 18,7 |
|  | 6 | 72 | 36,4 | 37,3 | 56,0 |
|  | 7 | 84 | 42,4 | 43,5 | 99,5 |
|  | Not sure | 1 | ,5 | ,5 | 100,0 |
|  | Total | 193 | 97,5 | 100,0 |  |
| Missing | System | 5 | 2,5 |  |  |
| Total | | 198 | 100,0 |  |  |

| **Q 2.19 The recommendations in this guideline are consistent with my patients’ values and preferences** | | | | | |
| --- | --- | --- | --- | --- | --- |
|  | | Frequency | Percent | Valid Percent | Cumulative Percent |
| Valid | 4 | 8 | 4,0 | 4,1 | 4,1 |
|  | 5 | 33 | 16,7 | 16,8 | 20,8 |
|  | 6 | 73 | 36,9 | 37,1 | 57,9 |
|  | 7 | 33 | 16,7 | 16,8 | 74,6 |
|  | Not sure | 50 | 25,3 | 25,4 | 100,0 |
|  | Total | 197 | 99,5 | 100,0 |  |
| Missing | System | 1 | ,5 |  |  |
| Total | | 198 | 100,0 |  |  |

| **Q 2.20 My patients do, or are likely to accept and follow the recommendations in this guideline** | | | | | |
| --- | --- | --- | --- | --- | --- |
|  | | Frequency | Percent | Valid Percent | Cumulative Percent |
| Valid | 3 | 1 | ,5 | ,5 | ,5 |
|  | 4 | 4 | 2,0 | 2,0 | 2,5 |
|  | 5 | 37 | 18,7 | 18,8 | 21,3 |
|  | 6 | 75 | 37,9 | 38,1 | 59,4 |
|  | 7 | 60 | 30,3 | 30,5 | 89,8 |
|  | Not sure | 20 | 10,1 | 10,2 | 100,0 |
|  | Total | 197 | 99,5 | 100,0 |  |
| Missing | System | 1 | ,5 |  |  |
| Total | | 198 | 100,0 |  |  |

| **Q 2.21 It is easy to find information in this guideline because the format and layout is easy to navigate** | | | | | |
| --- | --- | --- | --- | --- | --- |
|  | | Frequency | Percent | Valid Percent | Cumulative Percent |
| Valid | 3 | 1 | ,5 | ,5 | ,5 |
|  | 4 | 5 | 2,5 | 2,5 | 3,0 |
|  | 5 | 32 | 16,2 | 16,2 | 19,3 |
|  | 6 | 69 | 34,8 | 35,0 | 54,3 |
|  | 7 | 87 | 43,9 | 44,2 | 98,5 |
|  | Not sure | 3 | 1,5 | 1,5 | 100,0 |
|  | Total | 197 | 99,5 | 100,0 |  |
| Missing | System | 1 | ,5 |  |  |
| Total | | 198 | 100,0 |  |  |

| **Q 2.22 The wording of this recommendation is clear and unambiguous** | | | | | |
| --- | --- | --- | --- | --- | --- |
|  | | Frequency | Percent | Valid Percent | Cumulative Percent |
| Valid | 1 | 1 | ,5 | ,5 | ,5 |
|  | 2 | 2 | 1,0 | 1,0 | 1,5 |
|  | 3 | 2 | 1,0 | 1,0 | 2,6 |
|  | 4 | 13 | 6,6 | 6,6 | 9,2 |
|  | 5 | 42 | 21,2 | 21,4 | 30,6 |
|  | 6 | 81 | 40,9 | 41,3 | 71,9 |
|  | 7 | 48 | 24,2 | 24,5 | 96,4 |
|  | Not sure | 7 | 3,5 | 3,6 | 100,0 |
|  | Total | 196 | 99,0 | 100,0 |  |
| Missing | System | 2 | 1,0 |  |  |
| Total | | 198 | 100,0 |  |  |

| **Q 2.23 The guideline includes or is accompanied by implementation tools (clinician summary, patient summary, algorithm, medical record forms, etc.)** | | | | | |
| --- | --- | --- | --- | --- | --- |
|  | | Frequency | Percent | Valid Percent | Cumulative Percent |
| Valid | 1 | 3 | 1,5 | 1,5 | 1,5 |
|  | 2 | 5 | 2,5 | 2,5 | 4,1 |
|  | 3 | 8 | 4,0 | 4,1 | 8,1 |
|  | 4 | 11 | 5,6 | 5,6 | 13,7 |
|  | 5 | 35 | 17,7 | 17,8 | 31,5 |
|  | 6 | 53 | 26,8 | 26,9 | 58,4 |
|  | 7 | 28 | 14,1 | 14,2 | 72,6 |
|  | Not sure | 54 | 27,3 | 27,4 | 100,0 |
|  | Total | 197 | 99,5 | 100,0 |  |
| Missing | System | 1 | ,5 |  |  |
| Total | | 198 | 100,0 |  |  |

| **Q 2.24 Implementation tools included in or with the guideline (clinician summary, patient summary, algorithm, chart forms, etc.) are helpful to me, my practice or organization, or my patients** | | | | | |
| --- | --- | --- | --- | --- | --- |
|  | | Frequency | Percent | Valid Percent | Cumulative Percent |
| Valid | 2 | 1 | ,5 | ,5 | ,5 |
|  | 3 | 7 | 3,5 | 3,6 | 4,1 |
|  | 4 | 10 | 5,1 | 5,1 | 9,2 |
|  | 5 | 38 | 19,2 | 19,5 | 28,7 |
|  | 6 | 46 | 23,2 | 23,6 | 52,3 |
|  | 7 | 47 | 23,7 | 24,1 | 76,4 |
|  | Not sure | 46 | 23,2 | 23,6 | 100,0 |
|  | Total | 195 | 98,5 | 100,0 |  |
| Missing | System | 3 | 1,5 |  |  |
| Total | | 198 | 100,0 |  |  |

| **Q 2.25 The guideline is consistent with the available evidence** | | | | | |
| --- | --- | --- | --- | --- | --- |
|  | | Frequency | Percent | Valid Percent | Cumulative Percent |
| Valid | 3 | 1 | ,5 | ,5 | ,5 |
|  | 4 | 5 | 2,5 | 2,6 | 3,1 |
|  | 5 | 25 | 12,6 | 12,8 | 15,8 |
|  | 6 | 58 | 29,3 | 29,6 | 45,4 |
|  | 7 | 34 | 17,2 | 17,3 | 62,8 |
|  | Not sure | 73 | 36,9 | 37,2 | 100,0 |
|  | Total | 196 | 99,0 | 100,0 |  |
| Missing | System | 2 | 1,0 |  |  |
| Total | | 198 | 100,0 |  |  |

| **Q 2.26 The guideline describes whether patient preferences were collected and influenced the guideline questions, methods or recommendations** | | | | | |
| --- | --- | --- | --- | --- | --- |
|  | | Frequency | Percent | Valid Percent | Cumulative Percent |
| Valid | 1 | 2 | 1,0 | 1,0 | 1,0 |
|  | 2 | 9 | 4,5 | 4,6 | 5,6 |
|  | 3 | 7 | 3,5 | 3,6 | 9,2 |
|  | 4 | 14 | 7,1 | 7,2 | 16,4 |
|  | 5 | 10 | 5,1 | 5,1 | 21,5 |
|  | 6 | 18 | 9,1 | 9,2 | 30,8 |
|  | 7 | 9 | 4,5 | 4,6 | 35,4 |
|  | Not sure | 126 | 63,6 | 64,6 | 100,0 |
|  | Total | 195 | 98,5 | 100,0 |  |
| Missing | System | 3 | 1,5 |  |  |
| Total | | 198 | 100,0 |  |  |

| **Q 2.27 The guideline clearly describes underlying evidence supporting the recommendations** | | | | | |
| --- | --- | --- | --- | --- | --- |
|  | | Frequency | Percent | Valid Percent | Cumulative Percent |
| Valid | 1 | 1 | ,5 | ,5 | ,5 |
|  | 2 | 4 | 2,0 | 2,0 | 2,5 |
|  | 3 | 7 | 3,5 | 3,6 | 6,1 |
|  | 4 | 18 | 9,1 | 9,1 | 15,2 |
|  | 5 | 25 | 12,6 | 12,7 | 27,9 |
|  | 6 | 27 | 13,6 | 13,7 | 41,6 |
|  | 7 | 22 | 11,1 | 11,2 | 52,8 |
|  | Not sure | 93 | 47,0 | 47,2 | 100,0 |
|  | Total | 197 | 99,5 | 100,0 |  |
| Missing | System | 1 | ,5 |  |  |
| Total | | 198 | 100,0 |  |  |
